# Multimodal optical imaging with real-time projection of cancer risk and biopsy guidance maps for early oral cancer diagnosis and treatment

**DOI:** 10.1117/1.JBO.28.1.016002

**Published:** 2023-01-13

**Authors:** Jackson B. Coole, David Brenes, Ruchika Mitbander, Imran Vohra, Huayu Hou, Alex Kortum, Yubo Tang, Yajur Maker, Richard A. Schwarz, Jennifer Carns, Hawraa Badaoui, Michelle Williams, Nadarajah Vigneswaran, Ann Gillenwater, Rebecca Richards-Kortum

**Affiliations:** a Rice University, Department of Bioengineering, Houston, Texas, United States; b The University of Texas M. D. Anderson Cancer Center, Department of Head and Neck Surgery, Houston, Texas, United States; c The University of Texas M. D. Anderson Cancer Center, Department of Pathology, Houston, Texas, United States; d The University of Texas School of Dentistry, Department of Diagnostic and Biomedical Sciences, Houston, Texas, United States

**Keywords:** oral potentially malignant disorders, biopsy guidance, multimodal optical imaging, clinician guidance, high-resolution imaging

## Abstract

**Significance:**

Despite recent advances in multimodal optical imaging, oral imaging systems often do not provide real-time actionable guidance to the clinician who is making biopsy and treatment decisions.

**Aim:**

We demonstrate a low-cost, portable active biopsy guidance system (ABGS) that uses multimodal optical imaging with deep learning to directly project cancer risk and biopsy guidance maps onto oral mucosa in real time.

**Approach:**

Cancer risk maps are generated based on widefield autofluorescence images and projected onto the at-risk tissue using a digital light projector. Microendoscopy images are obtained from at-risk areas, and multimodal image data are used to calculate a biopsy guidance map, which is projected onto tissue.

**Results:**

Representative patient examples highlight clinically actionable visualizations provided in real time during an imaging procedure. Results show multimodal imaging with cancer risk and biopsy guidance map projection offers a versatile, quantitative, and precise tool to guide biopsy site selection and improve early detection of oral cancers.

**Conclusions:**

The ABGS provides direct visible guidance to identify early lesions and locate appropriate sites to biopsy within those lesions. This represents an opportunity to translate multimodal imaging into real-time clinically actionable visualizations to help improve patient outcomes.

## 1 Introduction

Oral cancer is a major global health issue with over 375,000 new cases reported per year.^1^ Early-stage diagnosis of oral cancer significantly reduces treatment-related morbidity and improves long-term survival; however, the worldwide 5-year survival rate of oral cancer patients is <50% because most patients are diagnosed at a late stage, when treatment is more invasive, more expensive, and less effective.^2^^,^^3^ Improving the early detection and treatment of oral cancer and its precursors offers the best opportunity to reduce the incidence and mortality of oral cancer.

Most oral cancers are preceded by clinically evident oral potentially malignant disorders (OPMDs) and microscopically evident altered epithelial changes known as oral epithelial dysplasia (OED). The current gold standard to diagnose OED is biopsy and histopathology of suspicious oral lesions that are identified by conventional visual oral examination.^4^^,^^5^ However, it can be difficult even for experienced clinicians to choose when and where to biopsy. Many general practitioners lack expertise to distinguish OPMDs from clinically similar benign lesions. This creates challenges to the clinicians in determining which oral lesions are at highest risk to contain OED and require biopsy.^6^^,^^7^

A variety of optical imaging approaches have shown promise to improve early detection of oral cancer and its precursors. Oral neoplasia is associated with a loss of blue-green autofluorescence (AF) due primarily to a decrease in the fluorescence of stromal collagen crosslinks (peak emission 470 nm at 405-nm excitation^8^) and an increase in red AF due to an increase in endogenous porphyrin fluorescence (peak emission 630 nm^9^).^10^ Oral neoplasia is also associated with changes in epithelial fluorescence associated with cofactors reduced nicotinamide adenine dinucleotide (NADH) (peak emission 450 nm) and flavin adenine dinucleotide (FAD) (peak emission >500  nm).^11^ The AF imaging can delineate areas containing high grade dysplasia and early oral cancer with improved sensitivity compared to clinical oral examination; however, specificity can be reduced due to loss of fluorescence associated with chronic inflammation.^12^^,^^13^

To provide high sensitivity and specificity, multimodal imaging strategies combining widefield and high-resolution imaging have been developed with a variety of techniques, including nonlinear optical microscopy,^14^ optical coherence tomography imaging with a forward-viewing probe,^15^ reflectance confocal microscopy,^16^ and fluorescence microscopy.^17^^–^^20^ While promising, these approaches suffer a number of challenges, including ensuring accurate coregistration between widefield and microscopic images; providing image analysis results in real time; and ensuring that clinicians can translate results of multimodal image analysis displayed on a computer monitor to a precise location of where to perform a mucosal biopsy. This is particularly challenging if the image features highlighting risk are not visible by the naked eye.

To overcome these limitations, we introduce the active biopsy guidance system (ABGS), a multimodal optical imaging and projection system and software architecture that fully integrates widefield AF imaging, microscopic imaging, and real-time projection of cancer risk maps. We demonstrate: (1) how cancer risk maps generated from widefield AF images can be used to guide the placement of the fiber optic probe for microscopic imaging of the oral epithelium, (2) that synchronized multimodal imaging can be used to register the anatomic locations where microscopic images were acquired, and (3) real-time integration of multimodal imaging to calculate and project a biopsy guidance map. Taken together, these results demonstrate the potential of the ABGS to integrate multimodal images and provide directly visible cancer risk and biopsy guidance maps to help clinicians improve the early detection of oral cancer and its precursors.

## 2 Methods

### 2.1 Instrumentation

**Fig. 1:**
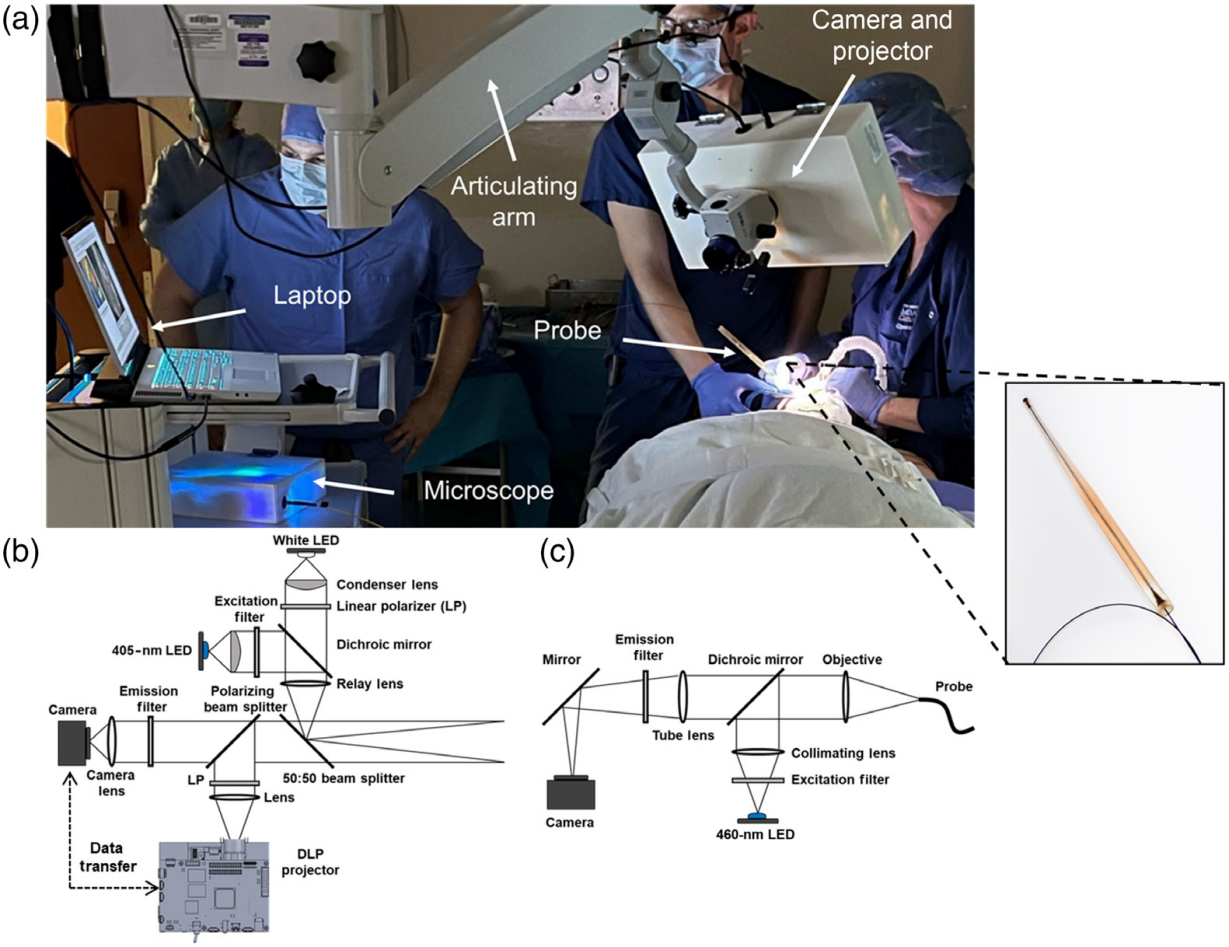
Optical subsystems and clinical implementation of the ABGS: (a) the ABGS in use in the operating room. The inset shows a zoomed in view of the microscope imaging probe. (b) Optical diagram of the ABGS camera and projector. (c) Optical diagram of the ABGS microscope. ABGS, active biopsy guidance system; LED, light emitting diode; DLP, digital light processing; and nm, nanometer.

The ABGS consists of two subsystems controlled through a single user interface: (1) a camera and projector for white light (WL) and AF image acquisition and real-time projection of visible risk maps and (2) a microscope with a fiber optic probe to image nuclear morphology.^21^ The ABGS is designed to guide a clinician through widefield and microscopic imaging to determine if and where a biopsy is needed for early detection of oral cancer and its precursors. Figure 1(a) shows a photograph of the ABGS system; the camera and projector are assembled and housed in a 3D-printed enclosure and mounted on a portable articulated arm. The microscope and laptop computer controlling the system are placed on a small cart. Cords connecting hardware to the laptop are run along the articulating arm. The system can be used in the operating room or an outpatient clinic.

Figure 1(b) shows a schematic of the camera and projector subsystem; a coaxial optical path enables a common field of view and focal plane for illumination, imaging, and projection. The camera can acquire WL and AF images; all optical components are assembled into a standard 30-mm optical cage system. The WL illumination is provided with a cool white illumination light emitting diode (LED) (Digi-Key, 1537-1193-ND) that is passed through a collimating lens (Thorlabs, ACL25416U) and linear polarizer (Thorlabs, LPVISE100-A). Illumination for AF imaging is provided by a 405-nm LED (Mouser, 897-LZ4V0UB0R00U8). Excitation light is passed through a collimating lens and a bandpass filter with peak transmission at 400 nm and a 40 nm full width at half maximum bandwidth (Semrock, FF01-400/40-25). A 425-nm dichroic mirror (Chroma, T425lpxr), relay lens (Thorlabs, LB1901), and beamsplitter (Thorlabs, BSW10R) are used to direct focused, coaxial WL and AF illumination to the imaging field of view. Reflected WL and returning AF pass through the beamsplitter and are directed through a linear polarizing beam splitter (Thorlabs, CCM1-PBS251), oriented with its axis of polarization perpendicular to the linear polarizer in the illumination path to reduce internal reflections. Returning signal passes through a 435-nm longpass filter (Omega, XF3088) and a collection lens focuses the image onto a complementary metal oxide semiconductor (CMOS) camera (FLIR, BFS-U3-23S3C-C) with a 1920×1200  pixel resolution that is downsampled to 960×600  pixels during multimodal imaging. The widefield camera and projector are designed to be used at a working distance of 35 cm from the edge of its enclosure and have a target field of view of 8×5  cm.

A digital light processing (DLP) module (Texas Instruments, LightCrafter4500) provides projection overlay capabilities. The projection lens at the end of the DLP light engine was removed as described in Scardigli et al.; the modified DLP projects a focused 10×16  mm image ∼15  mm from the projector.^22^ A relay lens (Edmund Optics, 49-767) is used to project this image onto the imaging field of view. Light from the projector is passed through a linear polarizer and then a polarizing beam splitter cube to align the projection with the optical axis of the camera and reduce specular reflection off the back surface of the cage system. Finally, the projection is transmitted through the beamsplitter that merges light from the illumination and imaging arms and is relayed onto the anatomical area of interest in the oral cavity.

The microscope component of the ABGS has been previously described^21^ [Fig. 1(c)]. Illumination is provided by a 460-nm LED (Mouser, 897-LZ1-10DB00-0100). Excitation light passes through an excitation filter (Edmund Optics, 84-705) and dichroic mirror (Chroma, AT485DC) and is focused onto the proximal surface of a coherent fiber optic bundle (Fujikura, FIGH-30-850N) that has 30,000 imaging cores with a center-center-spacing of <4  μm. Returning fluorescence is directed through a dichroic mirror and longpass filter (Semrock, FF01-550/88-25) and focused onto a CMOS camera (FLIR, BFS-U3-04S2M-CS). The fiber bundle is housed inside a 3D-printed holder as shown in the inset of Fig. 1(a). The microscope has a 790-μm-diameter field of view, 720×540  pixel resolution, and 4-μm lateral resolution and operates at 90 frames per second. The microscope is designed to image oral tissue following topical application of 0.01% proflavine, a topical fluorescent contrast agent that stains cell nuclei. The imaging probe is placed in gentle contact with the oral mucosa to image the size, shape, and distribution of epithelial cell nuclei.

### 2.2 Overview of Clinical Workflow

**Fig. 2:**
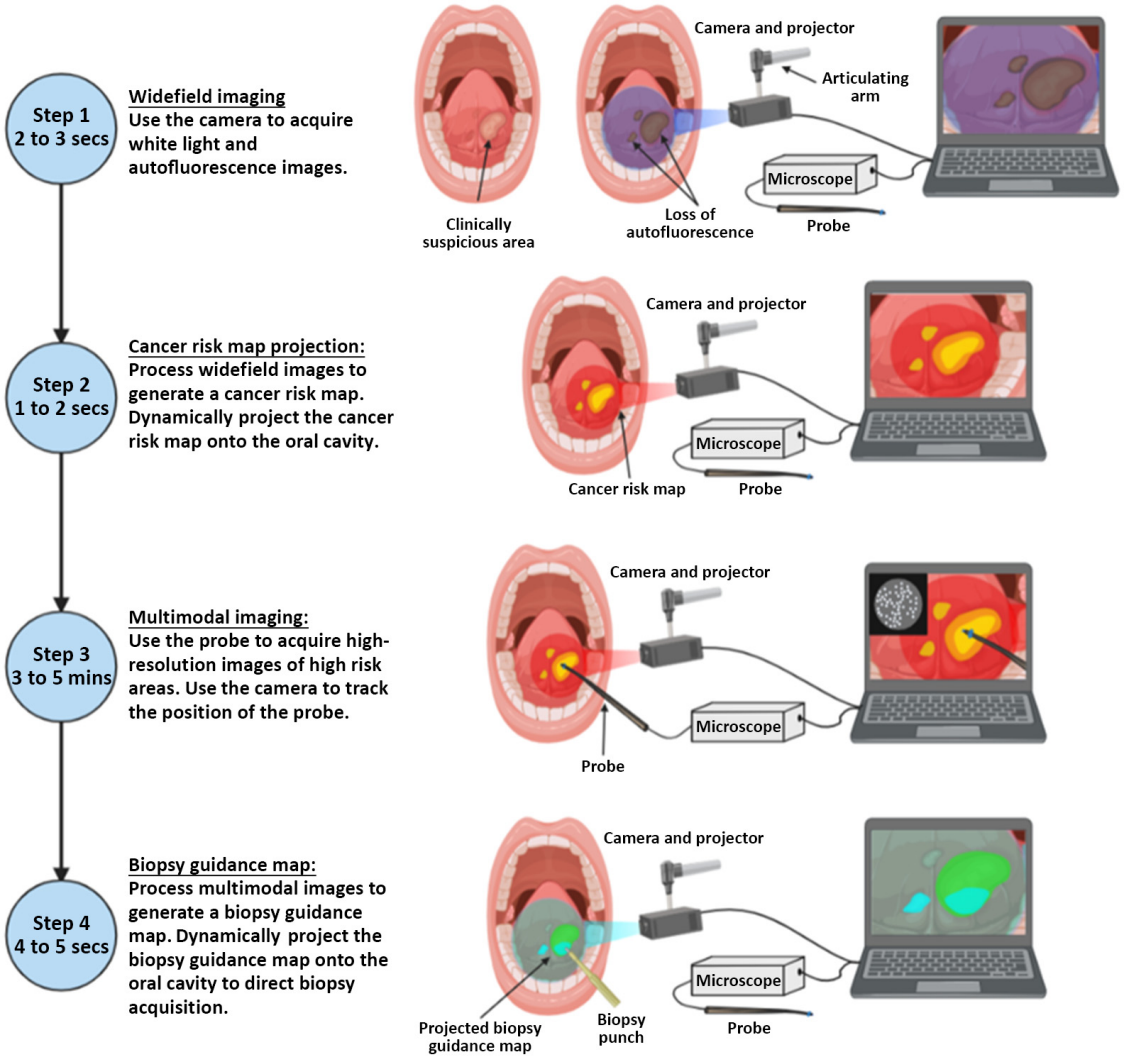
Imaging workflow of the ABGS. Step 1: widefield images are acquired using the camera and projector. Step 2: a cancer risk map is generated and projected onto the oral cavity. Step 3: multimodal images are acquired from the highlighted high-risk regions of tissue. Step 4: the multimodal image data are processed to generate and physically project a biopsy guidance map onto the oral cavity.

Figure 2 shows the workflow for biopsy guidance. In step 1, WL and AF images are acquired and displayed for the clinician’s reference. In step 2, a predictive cancer risk map is calculated from the AF image and is dynamically projected onto the oral mucosa of the patient; at the same time, the cancer risk map is overlaid on the AF image shown on the user interface. In step 3, the microscope imaging probe is used to acquire images from high-risk regions of tissue after application of a topical fluorescent contrast agent. Real-time video feeds from the camera and microscope are simultaneously saved and displayed on the user interface. In step 4, the coregistered multimodal images are processed to generate a biopsy guidance map that is dynamically projected onto the oral cavity.

### 2.3 Cancer Risk Map Generation and Projection

At the beginning of an imaging session, live video feed from the widefield camera is displayed and the articulating arm is adjusted until the lesion is in focus. The user presses a button to acquire a WL and AF image pair. The AF image is displayed and the user outlines the oral mucosa to exclude nonmucosal image regions (e.g., teeth, gloves, and retractors).

The cancer risk map is then generated and projected onto the oral mucosa. Previous studies have established that the normalized ratio of red-to-green (RG) fluorescence intensity can classify the presence of moderate dysplasia, severe dysplasia and cancer with high sensitivity and specificity.^13^^,^^17^ Here, a previously established algorithm was used to generate cancer risk maps from AF images;^23^ details can be found in Section S1.1 in the Supplementary Material. To physically project the cancer risk heatmap onto the oral mucosa, the DLP is treated as an additional display of the computer. A program resizes an image of the generated heatmap to the dimensions of the external monitor and casts the resized image in an external window to the DLP. Acquisition of the WL and AF images requires 2 s, and the cancer risk map is generated in less than 1 s.

### 2.4 Multimodal Imaging

The next step of the ABGS imaging workflow is to acquire microscopic images from areas identified as high risk based on widefield imaging. After projection of the cancer risk map, the clinician applies proflavine contrast agent to the oral mucosa with a cotton tipped applicator and directs the microscope imaging probe to areas highlighted as high risk for cancer. The system uses the widefield camera to track the position of the probe on the oral cavity during microscopic imaging. Live video feeds from the widefield camera and microscope are displayed and the clinician obtains microscopic images from any suspicious regions identified based on clinical impression or the projected cancer risk map.

Two image analysis processes run in the background during multimodal image acquisition: tracking the tip of the imaging probe to correlate microscopic images with the anatomic location where they were acquired, and diagnostic scoring of microscopic images. To facilitate probe tracking, colored dental ligatures are attached to the distal tip of the fiber holder. Color thresholding-based image analysis is used to segment the dental ligature and calculate the pixel coordinates of the distal tip of the microscope imaging probe. This tracking procedure can be run in real time for each widefield frame and the average positional error of tracking is <1.5  mm compared with manual hand annotations (Fig. S1 in the Supplementary Material). Real-time analysis of microscopic images is performed as described in (Fig. S2a in the Supplementary Material), and the diagnostic probability for the microscopic images is saved for images containing high quality morphometric information. Details describing the imaging probe tip tracking, multimodal image analysis, and real-time diagnostic scoring of microscopy images can be found in Sec. S1.2–S1.4 in the Supplementary Material. Details describing the electronics and software components that drive the ABGS can be found in Sec. S1.6 in the Supplementary Material.

### 2.5 Biopsy Guidance Map Generation and Projection

The final step in the ABGS imaging workflow is to process the multimodal imaging data to generate a biopsy guidance map and dynamically project it onto the oral cavity to direct biopsy acquisition. Immediately after multimodal imaging is finished, the custom software analyzes all the imaging data acquired as summarized in Figure S2b in the Supplementary Material. Because scoring the microscope images and probe tracking are performed in the background during the multimodal image acquisition, this data aggregation and real-time processing takes less than 5 s.

To generate biopsy guidance maps, the microscope diagnostic score generated by the multitask deep learning network (MTN) and RG ratio value are plotted for each pixel coordinate. Variable cutoff thresholds are manually and interactively set by the clinician during the imaging session for each parameter; any pixel with an RG ratio and MTN score both exceeding their respective thresholds is indicated for potential biopsy. To produce the biopsy guidance map, the location of any pixel in the image where both thresholds are exceeded is overlaid onto a blank canvas representing the field of view of the camera. The aggregate of all points overlaid on the canvas is saved as an image and projected onto the oral cavity. Once projected, two interactive slider bars on the user interface allow the biopsy guidance map to be updated in real time by adjusting the threshold values for the MTN scores and RG ratios. When a threshold is changed, a new biopsy guidance map is generated in less than a second by removing any values below the new threshold. This allows the user to interactively highlight specific areas of interest and to identify a precise location recommended for biopsy based on both loss of AF and changes in nuclear morphometry.

### 2.6 Patient Imaging

Imaging of human subjects with the ABGS was conducted at the University of Texas M. D. Anderson Cancer Center in Houston, Texas under an Institutional Review Board-approved protocol (M. D. Anderson protocol #2008-0613; Rice University protocol #2017-298). Patients with oral lesions scheduled to undergo surgical resection were eligible for enrollment in this study; informed written consent was obtained prior to imaging. Patients with an oral lesion were examined and their clinical management was determined by the clinician per standard of care. Each patient then underwent multimodal imaging with the ABGS prior to treatment as described in Fig. 2. After review of each imaging session, the clinician performed a biopsy or surgical resection per standard of care. Each biopsy was submitted for routine histopathologic analysis with subsequent review by the study pathologist.

## 3 Results

### 3.1 Widefield Imaging and Projection Resolution

**Fig. 3:**
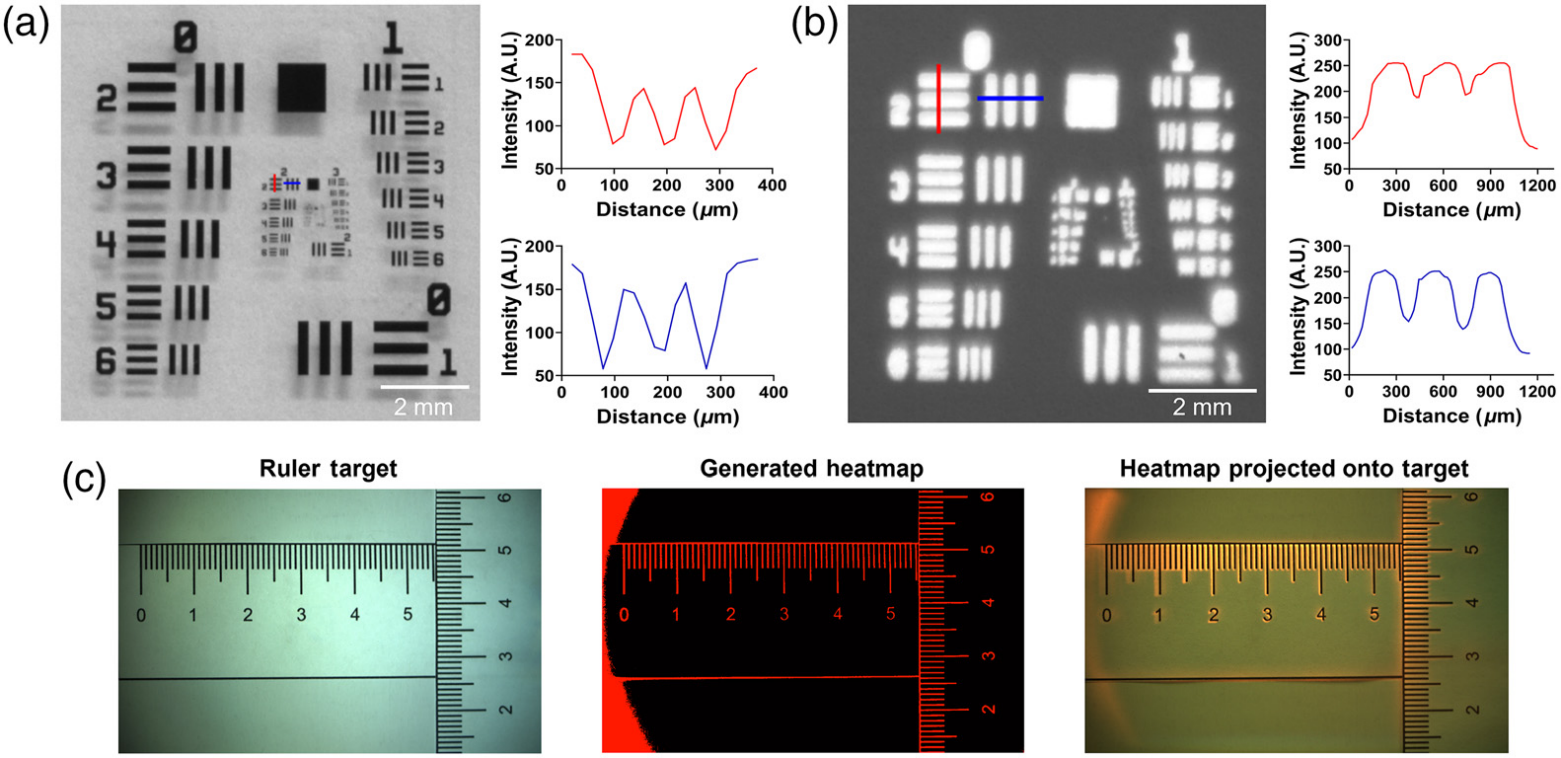
Evaluation of ABGS imaging and projection performance. The transverse optical resolution of the ABGS for imaging (110  μm) (a) and projection (400  μm) (b) measured by imaging a standard US Air Force calibration target and projecting the image of the calibration target onto a blank background. (c) The projection accuracy demonstrated by acquiring a WL image of a paper ruler target, generating a binary mask from the WL image, and projecting the binary mask back onto the ruler target.

To measure the spatial resolution of both the widefield imaging and projection components at the specified working distance, an image of a US Air Force resolution target was taken under WL illumination. This image was then projected back onto the same surface and an image of the projection was acquired to determine the resolution of projection. A line profile analysis shows that the system achieves a transverse resolution of 110  μm for imaging [Fig. 3(a)] and 400  μm for projection [Fig. 3(b)]. Further, to demonstrate coregistration of the fields-of-view of the camera and projection systems, a picture of a ruler was acquired. The image of the ruler was converted into a binary mask and projected onto the ruler; a second image of the ruler was acquired with the projection system on. The misalignment between the camera and projector was measured to be less than 1  mm [Fig. 3(c)].

### 3.2 Patient 1: Entire Clinical Workflow of ABGS

**Fig. 4:**
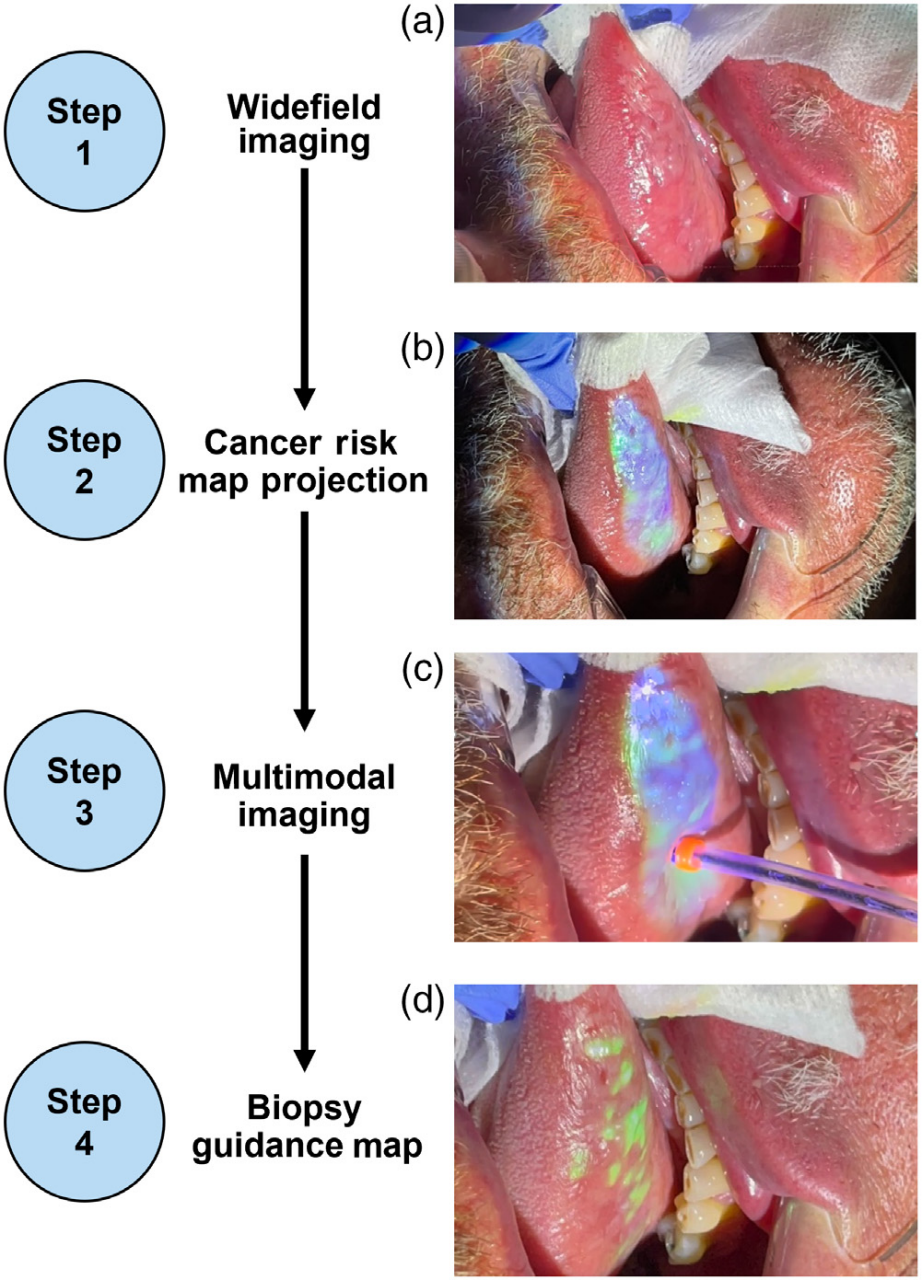
Clinician view during multimodal imaging. (a) View of the clinically suspicious area during widefield imaging. (b) View of the cancer risk map projected onto the oral cavity. (c) View of the imaging probe as it is translated across areas highlighted by the cancer risk map. (d) View of the final biopsy guidance map projected onto the oral cavity (Video 1, .mp4, 39.3 MB [URL: https://doi.org/10.1117/1.JBO.28.1.016002.s1]).

Figure 4 shows results throughout the ABGS imaging workflow on a patient presenting with a large (∼1.5 by 3 cm) heterogenous lesion with thin leukoplakia on the right lateral tongue (Video 1 in the Supplementary Material), highlighting what a clinician sees and does during an imaging procedure. First, a live feed of the widefield camera on the laptop display was used by the clinician to guide the placement of the widefield camera and projector so that the area of clinical suspicion was in focus [Fig. 4(a)].

Next, WL and AF images were acquired and displayed and the clinician outlined the oral mucosa. Once the mucosa was outlined, a cancer risk map was automatically generated and projected onto the lateral tongue [Fig. 4(b)]. The clinician applied proflavine to regions highlighted by the projected cancer risk map and multimodal image acquisition was initiated across all highlighted areas to obtain spatially registered microscopic images [Fig. 4(c)]. The live feed of the microscope and widefield camera were shown on the laptop display to provide real-time feedback on image quality; images were saved and analyzed in the background while the clinician translated the probe across the tissue. Immediately after multimodal imaging was finished, the data were automatically used to generate the biopsy guidance map that was physically projected onto the lateral tongue [Fig. 4(d)].

A detailed summary of the imaging steps, including representative widefield images, the cancer risk map, tracked locations of the imaging probe, and the biopsy guidance map can be found in Fig. S4 in the Supplementary Material. Briefly, a total of 3 min and 44 s of video were recorded during the multimodal imaging session, which included 15,731 microscope frames and 3410 widefield frames. Of the 15,731 total images, 5826 microscope frames passed both quality control steps and had a probability score generated and saved in real-time by the MTN. These probability scores, together with the RG ratios from the AF image, were used to generate a biopsy guidance map that was then projected onto the oral mucosa [Fig. 4(d)]. After the biopsy guidance map was projected onto the oral cavity, the clinician then determined suitable biopsy locations. The clinician obtained three biopsies from clinically suspicious regions; two biopsies (b2 and b3) were consistent with regions also identified by the biopsy guidance map. Biopsy location b1 was adjacent to an area highlighted by the biopsy guidance map. Histopathological analysis of all biopsies showed moderate to severe dysplasia.

**Fig. 5:**
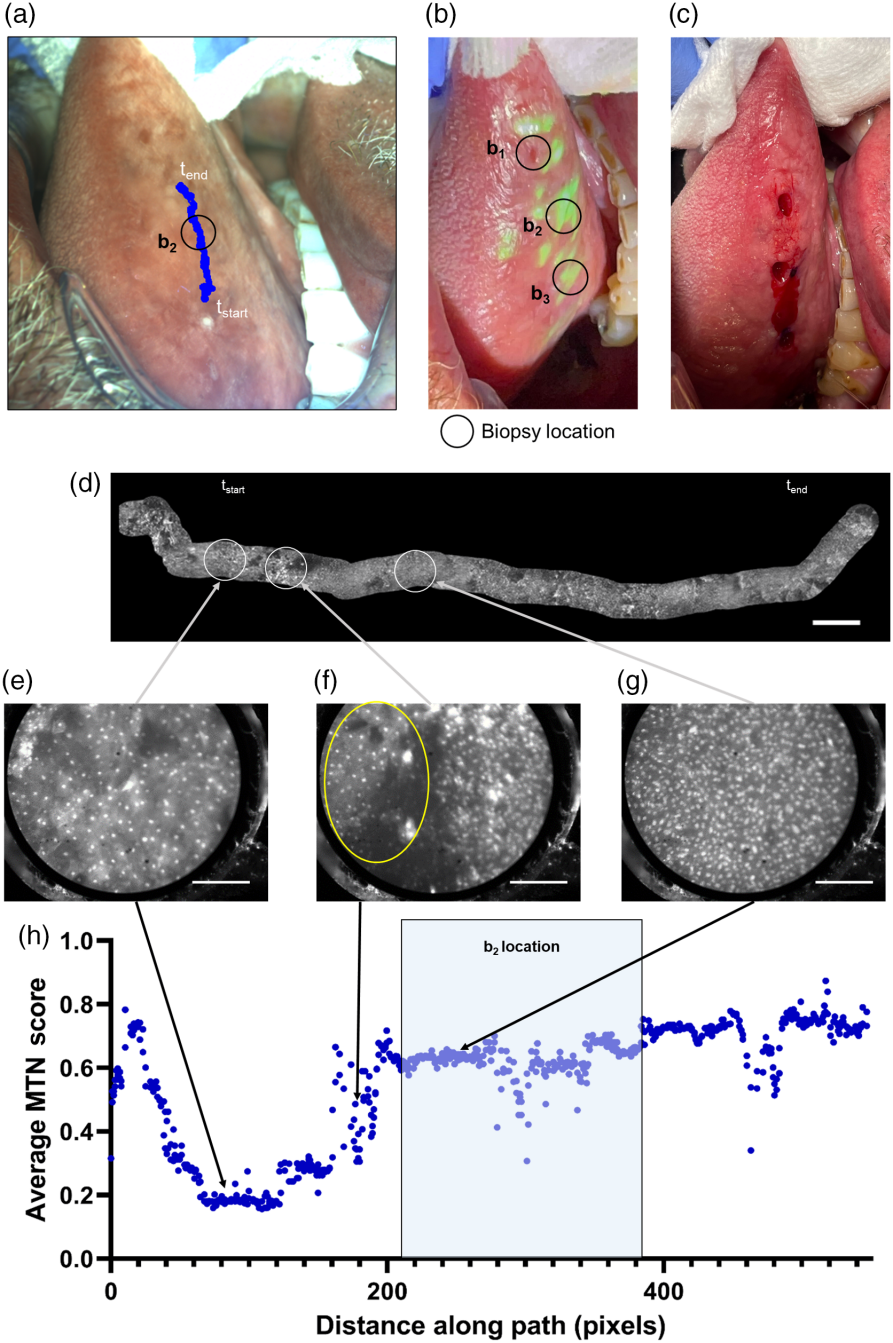
Analysis of multimodal imaging in a lesion in the right lateral tongue. (a) Representative WL image of the clinically suspicious area on the right lateral tongue annotated with the microscopic imaging path and location of the acquired biopsy. (b) WL image of the biopsy guidance map projected onto the oral cavity with the locations of all acquired biopsies overlaid. (c) Image of the acquired biopsies taken after multimodal imaging. (d) High-resolution mosaic generated from the multimodal imaging data with locations of the representative microscope images highlighted. (e) Microscope image showing regularly shaped and sparse nuclei characteristic of normal squamous epithelium. (f) Microscope image showing a transition zone between an area of regularly shaped and sparse nuclei (highlighted by the yellow oval) and an area of crowded, enlarged nuclei. (g) Microscope image showing crowded and enlarged nuclei. (h) Average MTN diagnostic score from microscope images versus distance along the imaging path. Scale bar on the high-resolution mosaic represents 1 mm, whereas scale bars on high-resolution images in panels (e)–(g) represent 200  μm. $t_{\text{start}}$, the starting location of the imaging session; $t_{\text{end}}$, the final location of the imaging session; MTN, multitask network; and $b_{1}$, biopsy 1.

To demonstrate the value of this coregistered dataset, a subset of data acquired during multimodal imaging was used to generate an image mosaic (Fig. 5). Widefield video shows the imaging probe as it is translated from the posterior lateral tongue toward the anterior area of the tongue; this sequence included 2100 microscope frames and 456 widefield frames. Pixel locations of the imaging probe were tracked and registered with each widefield imaging taken during the image sequence; these locations were then overlaid onto a representative WL image [Fig. 5(a)]. This overlay shows that the imaging path went directly through the location of the second biopsy (b2) acquired after multimodal imaging; this biopsy was acquired in an area that was suspicious by both clinical impression and the projected biopsy guidance map [Figs. 5(b)–5(c)]. The microscopic images from this subset were then analyzed and a high-resolution mosaic measuring 16.3 mm wide was generated [Fig. 5(d)].

Due to the high frame rate of the microscope, there can be many diagnostic scores associated with a single pixel location on the widefield image. Therefore, the average MTN score was calculated at each pixel. The average MTN scores along the mosaic and imaging path are shown in Fig. 5(h). The MTN scores highlight three distinct areas: a low-risk area where the MTN scores of microscopic images are low; a transition area where the MTN scores of the microscopic images rise; and a high-risk area where the MTN scores of the microscopic images remain high.

Representative images from these regions are shown in Figs. 5(e)–5(g). Figure 5(e) shows a microscopic image from the low-risk area with regularly shaped and sparse nuclei characteristic of normal squamous epithelium. As the probe was translated further upwards along the tongue, the MTN scores rise [Fig. 5(f)]. The nuclei highlighted inside the yellow oval in the left side of the microscope image are sparse, as is typical for benign areas of tissue, whereas nuclei in the right side of the image are more numerous and crowded, as is typically seen in dysplastic areas of tissue. After this transition, the remaining microscope images all have high diagnostic scores, and individual frames show crowded and enlarged nuclei [Fig. 5(g)]. The region of the mosaic with high MTN scores corresponds to an area highlighted for biopsy by both clinical impression and the projected biopsy guidance map [Fig. 5(b)]; histopathologic analysis showed moderate to severe dysplasia.

To further demonstrate the capability of the ABGS system, we show results from two additional patients with different kinds of lesions to illustrate generation and projection of the cancer-risk map and multimodal imaging.

### 3.3 Patient 2: Example of Widefield Imaging and Cancer Risk Map Projection

**Fig. 6:**
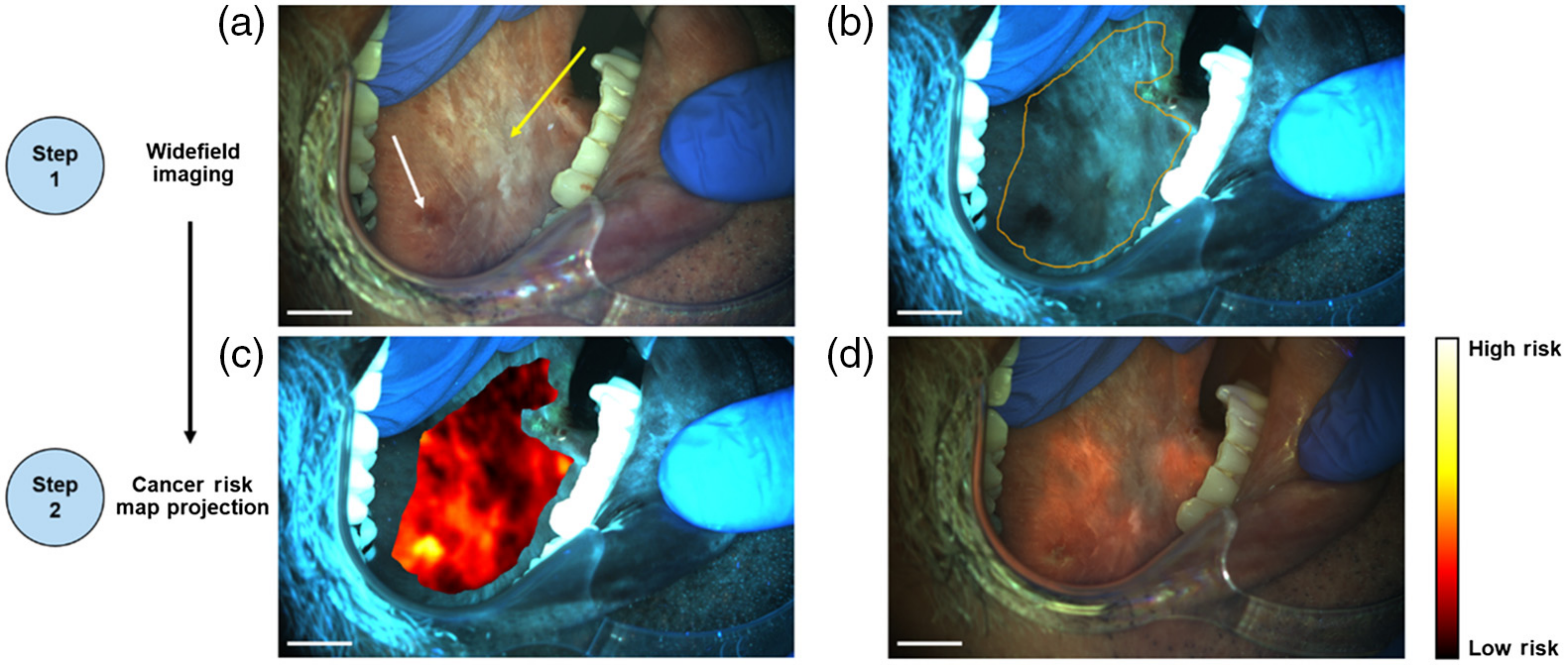
Cancer risk map generation and projection on a lesion in the right lateral tongue. (a) WL image. (b) AF image with mucosal boundary overlaid. (c) Cancer risk map overlaid onto the AF image. (d) Cancer risk map projected onto the oral cavity. Scale bars represent 1 cm.

Figure 6 shows an example of widefield imaging and cancer risk map projection in a second patient presenting with leukoplakia on the right lateral tongue. The widefield camera was focused on the lateral tongue and WL [Fig. 6(a)] and AF [Fig. 6(b)] images were acquired. The WL image shows a region of leukoplakia, appearing as a heterogeneous white patch [yellow arrow in Fig. 6(a)] and dark red tissue at the site of a previous biopsy in the bottom left of the image [white arrow in Fig. 6(a)]. The AF image showed loss of AF toward the posterior tongue, corresponding to the previous biopsy location and surrounding areas. The oral mucosa was then outlined, and the AF image processed to generate a cancer risk map. The cancer risk map, where higher risk corresponded to warmer colors, was overlaid on the AF image [Fig. 6(c)] and projected onto the oral mucosa of the patient [Fig. 6(d)]. This projected cancer risk map illuminated areas of highest risk that correspond to tissue exhibiting the largest loss of AF and is collocated with the lesion seen in Fig. 6(a), specifically the area surrounding the previous biopsy location.

### 3.4 Patient 3: Example of Multimodal Imaging

**Fig. 7:**
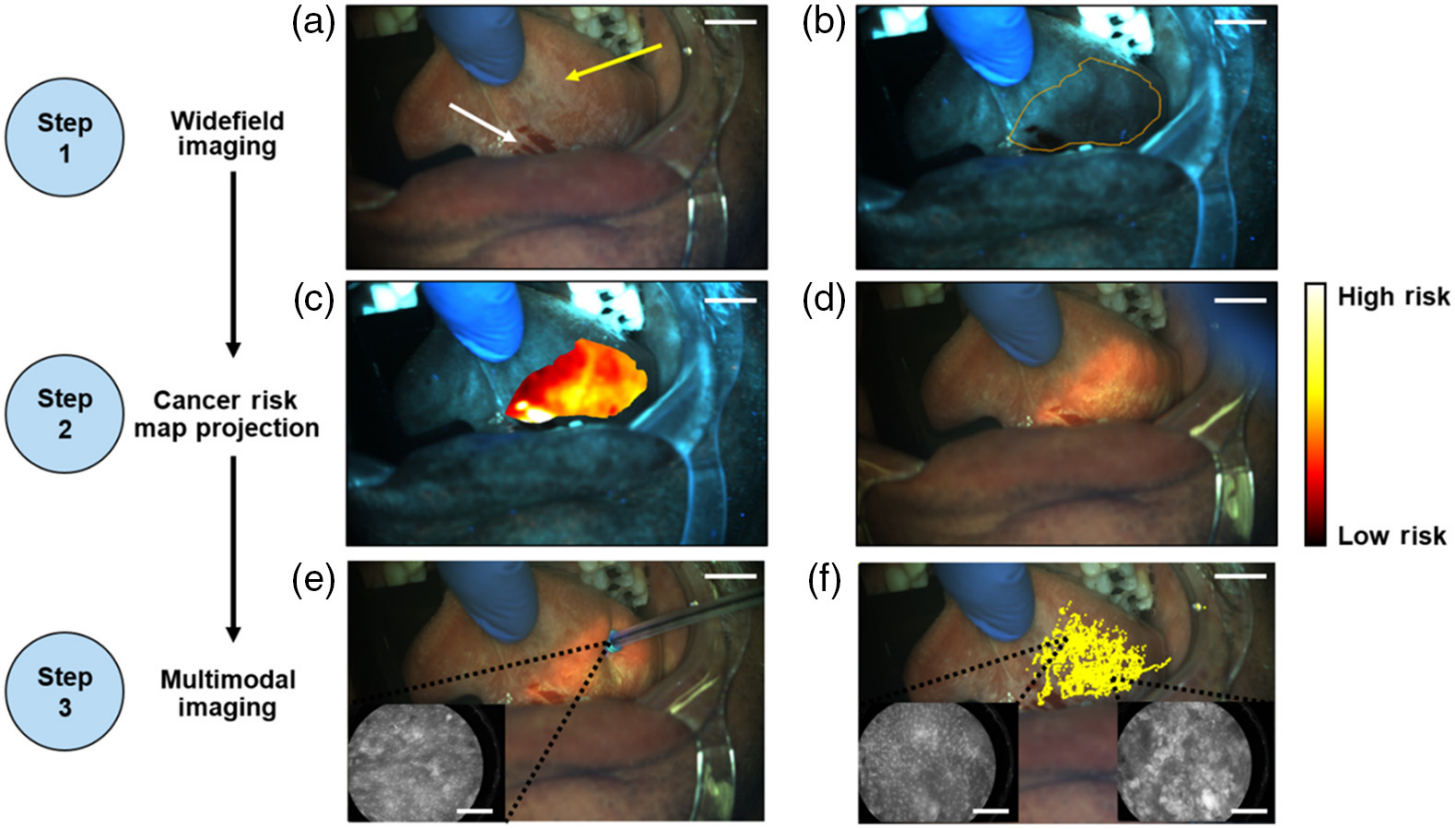
Multimodal imaging and probe tracking in a lesion in the left ventral tongue. (a) WL image. (b) AF image with mucosal boundary overlaid. (c) AF image with cancer risk map overlaid. (d) Cancer risk map projected onto the oral cavity. (e) Multimodal imaging to acquire microscopic images while tracking the probe position. (f) Tracked locations of the distal probe tip for each frame overlaid onto a representative WL image. Insets show example microscopic images and the corresponding locations from which they were acquired on the oral cavity. Scale bars represent 1 cm on widefield images and 200  μm on microscopic images.

Multimodal imaging was performed in a third patient presenting with leukoplakia on the left ventral tongue (Fig. 7). First, the widefield camera was used to acquire WL [Fig. 7(a)] and AF [Fig. 7(b)] images of the ventral tongue. The WL image showed a clinically suspicious area of leukoplakia [yellow arrow in Fig. 7(a)] next to an area that was bleeding from manipulation of the tongue during the procedure [white arrow in Fig. 7(a)]. The AF image showed loss of AF at the back of the ventral tongue and a dark area corresponding to the location of bleeding in the WL image. A cancer risk map was generated [Fig. 7(c)] and projected onto the oral mucosa [Fig. 7(d)].

Whereas the area of highest risk corresponds to the area of bleeding, the next highest areas of risk were in the inferior area of the ventral tongue below the clinically apparent lesion. This cancer risk map was used to guide placement of the microscope imaging probe to acquire microscopic images from high-risk areas while tracking the position of the probe [Fig. 7(e)]. In total, 4 min and 11 s of synchronized multimodal imaging was recorded, which included 22,642 microscope frames and 4579 widefield frames. Fiber tip tracking was performed in real time during the imaging session, and the location of the fiber tip in each widefield frame was overlaid onto the representative WL image [Fig. 7(f)]. Representative images and the locations they were acquired from in the oral cavity are shown in insets. The fiber tracking overlay demonstrates that the area highlighted by the cancer risk map was thoroughly sampled. The insets in Fig. 7(f) show two images acquired with the microscope. The image in the bottom left inset was acquired from a lower risk area and shows normal appearing nuclei characteristically seen with low grade areas. The image in the bottom right inset was acquired from a higher risk area and shows crowded nuclei characteristic of high-grade dysplasia. After multimodal imaging was performed, the clinician proceeded with clinical management per standard of care and surgically resected an area largely overlapping with the cancer risk map. Histopathological analysis of the specimen showed moderately differentiated invasive squamous cell carcinoma.

## 4 Discussion

This paper describes the ABGS, a multimodal optical imaging and projection system and software architecture that fully integrates widefield AF imaging, microscopic imaging, and real-time projection of cancer risk maps. This system is designed to generate cancer risk maps from widefield AF images to guide microscopic imaging of the oral epithelium with the fiber optic microscope probe, to use simultaneously acquired multimodal imaging to register microscopic images with the anatomical location from which they were acquired, and to integrate this multimodal imaging data to calculate and project a final biopsy guidance map. Imaging results from patients illustrate the potential clinical role of the ABGS in improving diagnostic precision and differentiation of OPMDs from clinically similar benign lesions, through the integration of multi-modal images and dynamic projection of cancer risk and biopsy guidance maps onto the oral cavity. This is critical for developing personalized treatment and follow-up for patients with OPMDs.

One key benefit of the ABGS is that it can physically project diagnostic information onto the field of view; this eliminates the need for a clinician to mentally transfer results of imaging from a computer display to the patient. For example, near-infrared fluorescence imaging has been extensively used for real-time surgical guidance of sentinel lymph node resection in several anatomical locations.^24^^,^^25^ These surgical guidance approaches typically combine the results of imaging into a digital overlay that highlights the anatomical area of interest in the field of view of the imaging device; this overlay is displayed on a computer monitor to the clinician during surgery and the clinician must mentally relay that information back onto the patient. Compared with these digital overlays, the physical projection of diagnostic information offers real-time actionable feedback without the risk of misregistration during the mental transfer from the display to the patient.

The design of the ABGS leverages consumer grade electrical and optical components to minimize cost and increase reproducibility; the microscope, imaging probe, and widefield camera and projector can be purchased and assembled for less than $9,000. While the ABGS was designed for blue light excitation fluorescence imaging to detect oral cancers, the multimodal imaging platform approach could be easily extended to other imaging modalities and anatomical areas of interest by combining the coaxial projection with alternative macroscopic optical approaches.

The high frame rate of the microscope and the large percentage of overlap between subsequent frames enables detection of subtle changes in cellular morphology at the surface of the epithelium. We have previously shown that this high frame rate combined with image analysis techniques can effectively increase the field of view of the microscope through the generation of high-quality mosaics.^21^ Whereas these large mosaics can be used to directly visualize large areas of the epithelium, previous implementations were unable to provide diagnostic guidance based on these generated mosaics. Here, using the MTN to score each microscope frame in a mosaic, it is possible to generate diagnostic predictions for specific areas within the mosaic. Further optimization is needed to leverage the diagnostic potential of these significantly increased fields of view provided by the high-frame-rate microscope.

It has previously been shown that combining widefield AF visualization with high-resolution point assessment of areas with abnormal AF can improve specificity.^17^^,^^18^ Yet, these algorithms were developed from a single representative image for a given anatomic site. Due to the vast increase in imaging data acquired using the ABGS system, further optimization is needed to leverage the significant increase in multimodal imaging information generated by the ABGS into robust multimodal algorithms.

## Code, Data, and Materials Availability

Data underlying the results presented in this paper are not publicly available at this time but may be obtained from the authors upon reasonable request.
