# Supplementary material for: Multimodal optical imaging with real-time projection of cancer risk and biopsy guidance maps for early oral cancer diagnosis and treatment
Source: J Biomed Opt. 2023 Jan 13;28(1):016002. doi: 10.1117/1.JBO.28.1.016002 (PMC9838568; doi:10.1117/1.JBO.28.1.016002)
Supplement: Supplementary file 1 [file JBO_028_016002_SD001.pdf]

Multi-modal Optical Imaging with Real Time Projection of Cancer Risk and Biopsy Guidance Maps for Early Oral Cancer Diagnosis and Treatment

Jackson B. Coole,[a] David Brenes,[a] Ruchika Mitbander,[a] Imran Vohra,[a] Huayu Hou,[a] Alex Kortum,[a] Yubo Tang,[a] Yajur Maker,[a] Richard A. Schwarz,[a] Jennifer Carns,[a] Hawraa Badaoui,[b] Michelle Williams,[c] Nadarajah Vigneswaran,[d] Ann Gillenwater,[b] Rebecca Richards-Kortum[a*]

[a]Rice University, Department of Bioengineering, Houston, Texas, United States
[b]The University of Texas M. D. Anderson Cancer Center, Department of Head and Neck Surgery, Houston, Texas, United States
[c]The University of Texas M. D. Anderson Cancer Center, Department of Pathology, Houston, Texas, United States
[d] The University of Texas School of Dentistry, Department of Diagnostic and Biomedical Sciences, Houston, Texas, United States

SUPPLEMENTARY FIGURES, METHODS, AND CAPTIONS

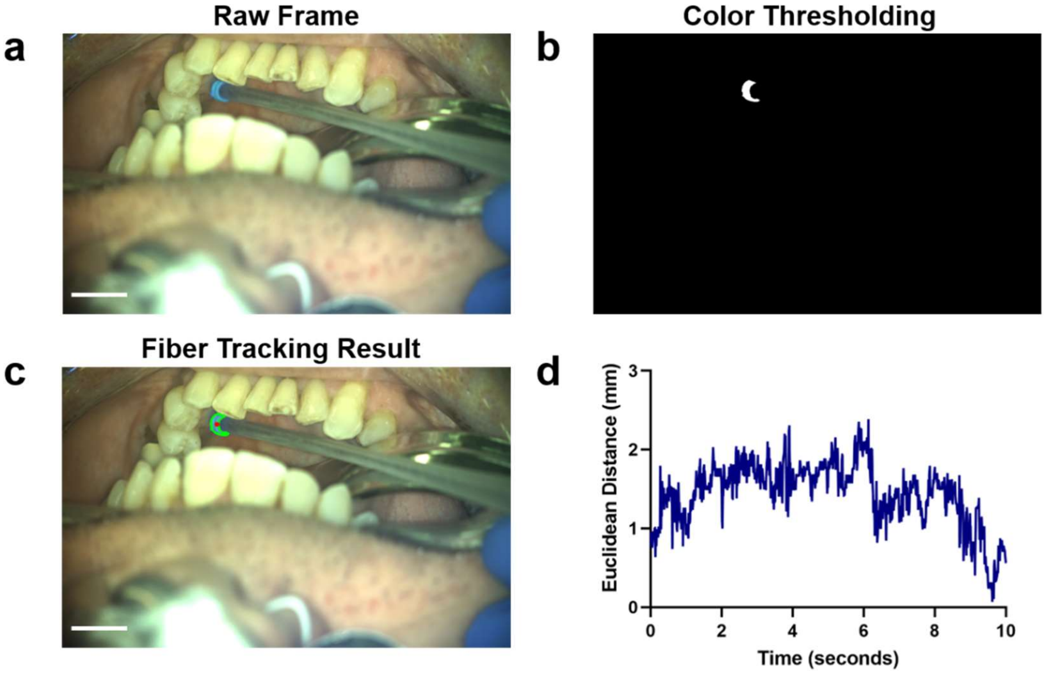

Fig. S1 Evaluation of fiber tracking performance in a patient with a gingival lesion in the left mandible. (a) A video sequence was acquired of the imaging probe being moved across the gingiva in the left mandible. (b) Each image of the video sequence was processed using color thresholding to determine the location of the colored bands on the 3D printed fiber holder. (c) Results of the color thresholding analysis were used to calculate the location of the tip of the fiber probe on the patient as designated by the red dot in the image. (d) The fiber tracking algorithm shows good accuracy; the Euclidean distance between the pixel coordinates of the fiber tip calculated by the automated tracking

algorithm and by hand annotations was <3 mm for all images in a nine second video sequence. Scale bars represent 1 cm.

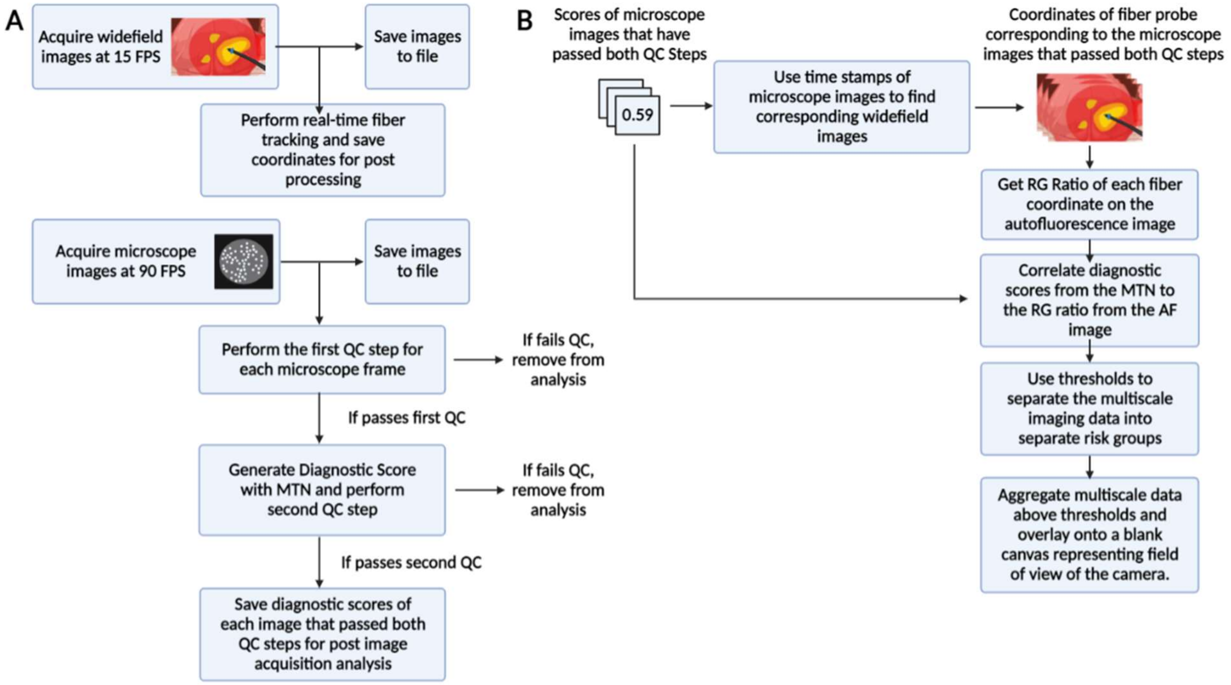

Fig. S2 Overview of real-time multi-modal image processing and Biopsy Guidance Map generation. (a) Overview of real time analysis of widefield and high-resolution microscope images during multi-modal image acquisition. (b) Overview of generation of the Biopsy Guidance Map immediately after multi-modal image acquisition is finished. Acronyms: FPS – Frames per second; QC – Quality Control; MTN – Multi-task Network; RG – Red to Green.

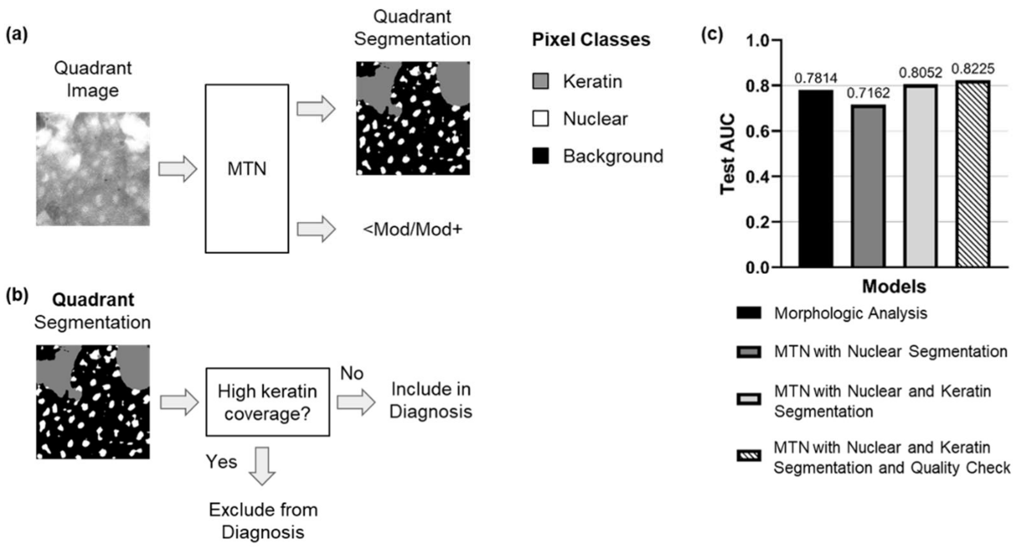

Fig. S3 High resolution image processing. (a) Oral HRME quadrant image is processed by the MTN to generate the 3-class quadrant segmentation (keratin, nuclear, and background segmentation) and predict pathology. (b) The generated segmentation mask is used to determine the degree of keratin coverage. Quadrants with a high keratin coverage are excluded from diagnosis. (c) Test set area under the receiver operating curve (AUC) for detection of Mod+ disease of the MTN model variants and the morphologic analysis algorithm. Tested MTN variants include MTN with nuclear segmentation only, MTN with nuclear and keratin segmentation, and a MTN with nuclear and keratin segmentation with quality check based on keratin coverage. Acronyms: MTN – Multi-task Network; Mod+ - pathology grade moderate dysplasia or more severe.

Histopathology	Training	Validation	Test
No Dysplasia	6 (182)	5 (93)	5 (93)
Mild Dysplasia	14 (116)	9 (78)	6 (57)
Moderate Dysplasia	14 (90)	9 (32)	5 (33)
Severe Dysplasia	11 (72)	7 (43)	4 (28)
Cancer	39 (197)	23 (93)	17 (101)

Table S1 Number of patients and number of microscope images in the training, validation, and test sets of the oral MTN (Multi-Task Network) stratified by patient histopathologic diagnosis. Number of microscope images are in parentheses.

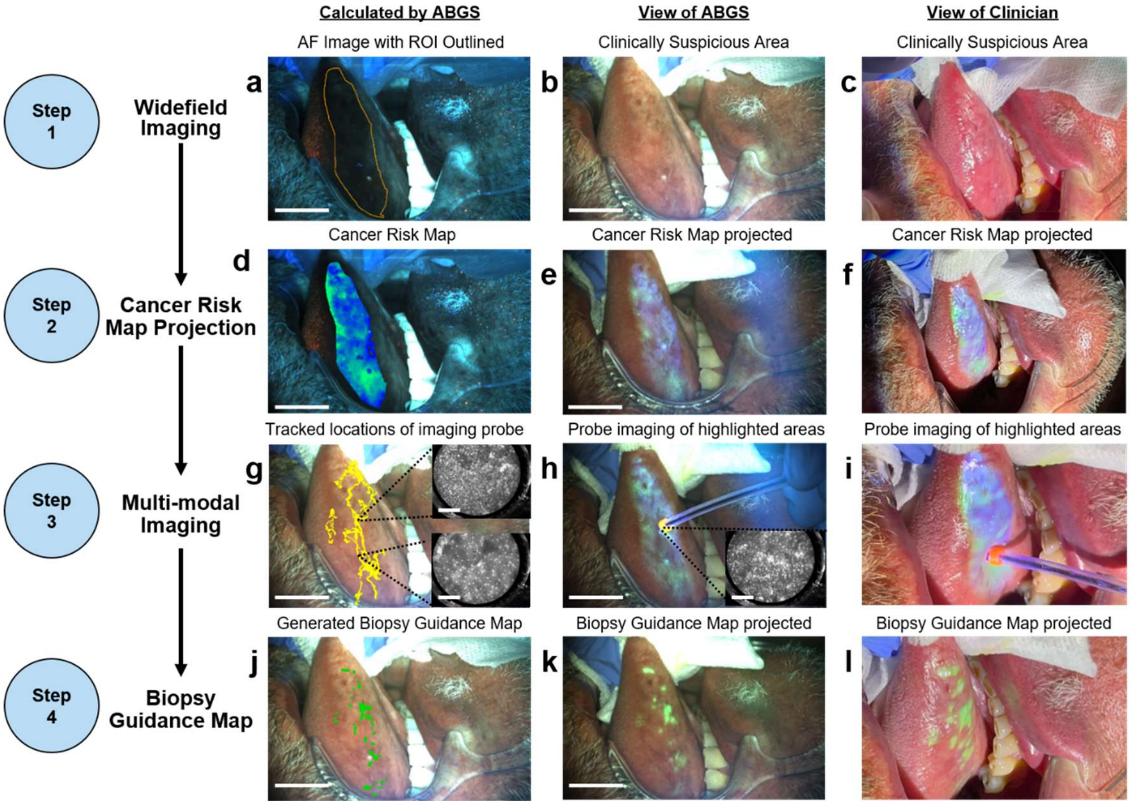

Fig. S4 Multi-modal imaging and analysis from a lesion on the right lateral tongue. ABGS acquired (a) autofluorescence and (b) white light images of clinically suspicious area. (c) Clinician's view as captured by a cell phone camera. (d) Cancer risk map overlaid on the AF image, (e) imaged by the ABGS, and (f) viewed by the clinician. (g) ABGS tracked locations of the high-resolution probe overlaid on the white light image. (h) ABGS view of probe acquiring high resolution images from areas indicated by the projected cancer risk map and (i) as viewed by the clinician. (j) Biopsy guidance map overlaid on WL image, (k) biopsy guidance map projected on the tissue as viewed by the ABGS (l) and as viewed by the clinician. Scale bars on widefield images represent 1 cm while scale bars on the high-resolution images represent 200 um. Acronyms: AF – Autofluorescence; ROI –Region of interest; ABGS – Active Biopsy Guidance System.

2 Supplemental Methods

S1.1 Cancer Risk Map Generation and Projection

Cancer risk maps are produced by calculating the normalized red-to-green fluorescence intensity ratio at each pixel in the AF image. First, the pixel intensity values of the red and green channels of an autofluorescence image are used to calculate the ratio of red fluorescence to that of green fluorescence in each pixel. The resulting red-to-green intensity ratio is then normalized by the smallest red-to-green intensity ratio in the image, which represents a region of normal tissue. The normalized red-to-green fluorescence intensity ratio is then converted into a heatmap using a look-up table corresponding to a standard color map. Pixels with a normalized RG ratio below a minimum threshold are not assigned a color and are excluded from the heatmap, whereas pixels with an RG ratio above a maximum threshold are assigned the highest value of the look up table. The remaining pixel values with a normalized RG ratio between the minimum and maximum thresholds were distributed into the chosen color map. Thresholds were set based on results of previous studies [17], [23] and were used prospectively in this study.

S1.2 Imaging Probe Tip Tracking

Colored dental ligatures were attached to the distal tip of the imaging probe in order to track location of the probe tip as high-resolution imaging is performed. Multiple bands of the same color were placed next to one another to assist in image analysis. Fiber tracking was accomplished using hue, saturation, and value (HSV) based color thresholding. Morphological filtering was applied to the segmented pixels to remove noise and then the centroid of the largest group detected was identified as the fiber tip. This pixel location of the fiber was recorded in real-time for each image.

S1.3 Multi-modal Imaging Analysis

The multi-modal image analysis pipeline is outlined in Supplemental Fig. S2b. After multi-modal imaging is finished, a background processing step synchronizes the two imaging feeds together. Due to the difference in frame rates of the widefield camera (30 FPS) and the microscope (90 FPS), it is first necessary to correlate the two video feeds together before further analysis. Since the microscope operates at a higher frame rate, there are several microscope images that correspond to a single widefield image. This analysis generates a table with the name of the microscope image and its corresponding widefield image. Synchronized videos are generated from this table by overlaying the microscope image onto its corresponding widefield frame.

Next, the program aggregates all imaging data acquired during the session, including imaging probe locations, microscope image scores, and normalized red-to-green fluorescence intensity ratios at each location imaged. This aggregated information is used to generate the final biopsy guidance map that is dynamically projected onto the oral cavity of the patient. During this analysis, the coordinates of the tracked imaging probe tip are correlated to each widefield image to determine the anatomic location from which each high resolution image was acquired. A diagnostic score is assigned at the location of the high resolution imaging probe in each widefield image; the assigned score is computed as the average of the diagnostic scores of all microscope images corresponding to that single widefield image. Finally, the RG ratio is calculated at the position of the tracked imaging probe tip in the autofluorescence image. After this multi-modal correlation, the aggregated multi-modal imaging data includes the name of the widefield image, the pixel location of the imaging probe in that widefield image, the average diagnostic score of the microscope images taken at that location, and the RG ratio value from the autofluorescence image at that pixel location.

S1.4 Real-time diagnostic scoring of microscopy images

A custom image analysis pipeline was created to allow for real-time diagnostic scoring of microscopy images by deploying the multi-task network in a python executable that runs in the background of the main user interface. Immediately after image acquisition, the microscope image undergoes an initial quality assurance check to determine if the imaging probe was in firm contact with tissue (i.e. nuclei are visible and in focus) and that the image quality was sufficient for diagnostic scoring. If the microscope image passes the first check, the image is saved to file and sent to the multi-task network for diagnostic scoring. During multi-modal image acquisition, the python program polls the list of all acquired microscope images and scores the microscope image most recently acquired to ensure representative sampling throughout the imaging procedure. As described above, the microscope image is segmented to identify nuclei and regions corresponding to keratin and scored. The segmentation procedure is used as a second quality control metric; images that contain predominantly keratin or too few nuclei do not pass this quality control review and are removed from further analysis. The diagnostic scores generated by the multi-task network for images passing quality review are saved to a data table for multi-modal analysis. Using this pipeline, microscope images can be analyzed and scored at a speed of ~30 FPS.

S1.5 Multi-task Network Training and Validation

We have previously shown that automated image analysis algorithms can distinguish cancer and high-grade dysplasia from benign tissue with high accuracy in a variety of anatomical sites including the cervix and oral cavity [18,26,27], and that incorporating quality control metrics into these algorithms can further improve performance [28]. This approach was translated into a recently described deep learning framework called the multi-task deep learning network (MTN)

to detect cervical precancer and cancer [29]. For this study, we developed and deployed a new MTN trained with high resolution imaging data taken from the oral cavity.

The MTN used in this study was developed using microscope images collected from a total of 174 patients undergoing surgical resection at the University of Texas M. D. Anderson Cancer Center under a previous patient imaging protocol described by Pierce et al. and Quang et al [17], [18]. In that study, patients undergoing surgery for oral dysplasia or cancer were imaged in the operating room immediately prior to surgery. The surgeon selected microscope imaging sites based on their clinical impression and acquired microscope videos from all sites of interest. Punch biopsies were collected according to the standard of care and pathology results were correlated with corresponding microscope videos. Then, each video was separated into individual frames and each frame was labeled with the histopathological ground truth of the anatomical site imaged. Data from that previous study were stratified by pathologic diagnosis and partitioned into training, validation, and test sets using a 50:30:20 split as shown in Table S1. For each image, a mask denoting nuclear and keratin pixels was created. Nuclear pixels were identified by a previously validated, automated nuclear segmentation algorithm for microscopy image analysis [29]. Keratin pixels were manually identified by three raters; their annotations were combined via majority voting. The final semantic segmentation mask was created by superimposing the keratin mask onto the nuclear mask.

The MTN was trained in two stages. Stage one independently optimized the semantic segmentation component. Performance was evaluated after every training step on the validation set using mean intersection over union (mIOU). In stage two, the full MTN architecture was trained. The semantic segmentation component was initialized with weights learned in stage one and the classification component was randomly initialized. The MTN's classification component

was trained to differentiate between <Mod and Mod+ sites. Performance was evaluated after each training step on the validation set using area under the receiver operating curve (AUC). The model with the highest validation AUC was evaluated on the test set. The MTN performance was compared to a previously validated morphologic analysis algorithm [29], and to three variants of the MTN: 1) a MTN with nuclear segmentation only; 2) a MTN with nuclear and keratin segmentation; and 3) a MTN with nuclear and keratin segmentation that incorporated an internal quality check based on keratin coverage (Supplemental Fig. S3c). The version of the MTN that incorporated the nuclear and keratin segmentation as well as an internal quality check based on keratin coverage performed the best and was used for this study.

During multi-modal imaging, microscope images are cropped to isolate the inner portion of the fiber bundle and split into four quadrants that are each processed independently by the MTN. The MTN performs two tasks: semantic segmentation and classification (Supplemental Fig. S3a). Semantic segmentation is performed by an encoder-decoder module with skip connections. During semantic segmentation, pixels in the image quadrant are categorized into three groups: nuclear, keratin, or background. The percentage of the quadrant categorized as keratin is calculated, and quadrants with a high keratin coverage are excluded from diagnosis (Supplemental Fig. S3b). Classification is performed by a second encoder that uses features generated by the semantic segmentation encoder as input. The classification component generates a value between 0 and 1 that corresponds to the probability that quadrant originates from a site with pathology grade moderate dysplasia or more severe disease (Mod+). To obtain the final microscope image score, the Mod+ probabilities of each quadrant with sufficient high nuclear pixel content are averaged.

S1.6 ABGS Software and Hardware Integration

Software to perform multi-modal image acquisition and analysis was developed in LabVIEW 2021 (National Instruments, Austin, TX, USA) and run on a laptop computer. A user interface was created to integrate multi-modal acquisition and real-time display of the ABGS imaging feeds, real-time analysis of autofluorescence images to calculate cancer risk heatmaps, real-time scoring of microscope images to calculate biopsy guidance maps, and projection of cancer risk and biopsy guidance maps. This user interface also guides the clinician through the imaging workflow.

The hardware and electronics of the microscope component of the ABGS has been previously described [16]. The optical assembly of the widefield imaging component of the ABGS is secured inside a 3D printed housing. All internal hardware including the CMOS camera, the microcontroller, and the DLP is connected to a single USB hub inside of the encasement. This hub is connected to a male-to-male adapter (part #) bridging the inside and outside of the 3D printed enclosure, and finally to the laptop computer through USB cables.

The DLP and the electronic circuitry powering the LEDs for white light and blue light illumination are both powered by a single 12 V power supply (217-2930-ND, Digi-key). The LED illumination circuitry is controlled through a custom built PCB that has connections for the power supply, two LEDs, an Arduino Nano microcontroller, resistors (two 68 Ohm and two 6.8 kOhm), two transistors (Digi-key,497-STX13003-APCT-ND), and a 1000mA BUCKPUCK LED Driver (3021-D-E-1000, Digi-Key) that regulates the 12 V power supply for the LEDs. During image acquisition, the white light and blue light LEDs are programmatically turned on and off through the LabVIEW user interface by using functions provided by the LabVIEW MakerHub LINX toolkit. This toolkit allows LabVIEW to interface with the Arduino Nano soldered onto the PCB,

and sends analog commands from the microcontroller to the CTL pin of the LED driver. This allows for the LED brightness to be modulated or for the LED to be turned on or off.

S1.7 Mosaic Generation

Image mosaics with large fields of view can be assembled from sequentially acquired high-resolution images; robust tracking of the imaging probe during image acquisition allows precise spatial co-registration of a mosaic with specific anatomic locations in the oral cavity. Thus, transitions in the morphology of epithelial cell nuclei and the corresponding probability of high-grade dysplasia can be visualized along the imaging path. High resolution mosaics were generated using a previously described approach [16]. Briefly, the region of the image corresponding to the active area of the fiber bundle is identified and all frames are downsampled to remove the fiber bundle pattern from the image. Then, an AKAZE feature detector is used to perform a rigid registration between each successive pair of frames, and a pixel shift representing the relative translation between the two frames is calculated. Finally, imaging frames are inserted sequentially into a blank canvas based on the calculated pixel shifts.
